# Protective effects of multi-strain probiotics against polystyrene nanoplastic-induced parotid gland toxicity: role of NRF2/HO-1 and NF-κB pathways

**DOI:** 10.3389/fphar.2026.1824052

**Published:** 2026-05-25

**Authors:** Günay Kozan, Hilal Üstündağ, Adem Kara

**Affiliations:** 1 Department of Ear, Nose Throat, Faculty of Medicine, Dicle University, Diyarbakır, Türkiye; 2 Department of Physiology, Faculty of Medicine, Erzincan Binali Yıldırım University, Erzincan, Türkiye; 3 Department of Molecular Biology and Genetics, Faculty of Science, Erzurum Technical University, Erzurum, Türkiye

**Keywords:** inflammation, nanoplastics, Nrf2 pathway, oxidative stress, parotid gland, polystyrene, probiotics

## Abstract

**Background:**

Polystyrene nanoplastics (NPs) represent emerging environmental contaminants with potential adverse effects on salivary glands. This study investigated the protective effects of multi-strain probiotics against NPs-induced toxicity in rat parotid glands.

**Methods:**

Forty male Sprague-Dawley rats were allocated into five groups (n = 8): control, NPs (10 mg/kg), probiotics (10^10^ CFU), NPs + probiotics low dose (10^9^ CFU), and NPs + Pro high dose (10^10^ CFU). Treatments were administered orally for 35 days. Oxidative stress markers, histopathological changes, and protein expression were evaluated.

**Results:**

NPs exposure significantly increased MDA levels (p < 0.05) and decreased SOD activity and GSH content (p < 0.05). Histopathological examination revealed moderate to severe acinar degeneration (++) and fibrosis (++) in NPs-treated animals. Western blot analysis showed significant downregulation of Bcl-2 (p < 0.01), HO-1 (p < 0.0001), NRF2 (p < 0.01), and AQP5 (p < 0.0001), with upregulation of Caspase-3 (p < 0.0001), NF-κB (p < 0.001), IL-1β (p < 0.001), and TNF-α (p < 0.001). Probiotic supplementation significantly ameliorated oxidative stress (p < 0.05), reduced histopathological damage to mild changes (+), and restored beneficial protein expression while suppressing inflammatory markers (p < 0.01-0.001).

**Conclusion:**

This study provides the first evidence that NPs induce significant oxidative stress and tissue damage in parotid glands. Multi-strain probiotics provide substantial protection by activating the NRF2/HO-1 pathway and suppressing inflammation, suggesting a promising therapeutic strategy against nanoplastic-induced oral tissue damage.

## Introduction

Plastic pollution has emerged as one of the most pressing environmental challenges of the 21st century, with global plastic production reaching approximately 460 million tons annually and projected to reach 1.2 billion tons by 2060 (Thompson et al., 2009). The inadequate disposal of plastic waste and its persistence have become a serious challenge to the environment, health, and wellbeing of living creatures, including humans (Khanna et al., 2024). Due to limited biodegradation capabilities, plastic materials accumulate in various environmental compartments and undergo progressive fragmentation through physical, chemical, and biological processes, ultimately generating microplastics (MPs; 1 μm-5 mm) and nanoplastics (NPs; <1 μm) (Winiarska et al., 2024).

Microplastics and nanoplastics represent emerging environmental contaminants of particular concern due to their ubiquitous distribution across terrestrial, aquatic, and atmospheric environments (Leslie et al., 2022). These particles have been detected throughout the human body, including blood, saliva, liver, kidneys, placenta, and even brain tissue, raising significant concerns about their potential health implications (Marfella et al., 2024). The average person is estimated to ingest more than 200,000 microplastic particles annually through contaminated food and water, with additional exposure via inhalation and dermal contact (Mani, 2024).

NPs are of particular toxicological significance due to their enhanced ability to penetrate biological barriers and accumulate within cells and tissues (Peng et al., 2023). Recent studies have demonstrated that these particles can trigger various pathological processes, including oxidative stress, chronic inflammation, DNA damage, and endocrine disruption (Deng et al., 2025). The mechanisms underlying NPs’ toxicity involve disruption of cellular homeostasis, mitochondrial dysfunction, and activation of pro-inflammatory cascades, ultimately leading to tissue damage and organ dysfunction (Casella and Ballaz, 2024).

Salivary glands are critical components of the oral health ecosystem, producing 0.5–1.5 L of saliva daily to facilitate mastication, swallowing, speech, and digestion, while providing antimicrobial protection (Proctor and Carpenter, 2014). The parotid glands, being the largest of the major salivary glands, are particularly vulnerable to environmental toxins due to their high metabolic activity and direct exposure to ingested contaminants (Carpenter, 2013). These glands play essential roles in maintaining oral homeostasis by producing serous saliva that contains enzymes, electrolytes, and antimicrobial compounds that protect against bacterial infection and support digestive processes (Amerongen and Veerman, 2002).

Oxidative stress has been identified as a primary mechanism of NPs-induced cellular damage, characterized by an imbalance between reactive oxygen species production and antioxidant defense systems (Rahman and MacNee, 2000). In salivary glands, oxidative stress disrupts normal secretory function, compromises cellular integrity, and triggers inflammatory responses that can lead to glandular dysfunction and tissue degeneration (Nagler, 2004). The nuclear factor erythroid 2-related factor 2 (NRF2) pathway serves as the master regulator of cellular antioxidant defense, controlling the expression of numerous cytoprotective genes, including heme oxygenase-1 (HO-1), superoxide dismutase (SOD), and glutathione synthesis enzymes (Yamamoto et al., 2018).

Probiotics (Pro), defined as live microorganisms that confer health benefits when administered in adequate amounts, have gained significant attention for their potential protective effects against environmental toxins (Hill et al., 2014). These beneficial bacteria exert their effects through multiple mechanisms, including immune system modulation, enhancement of antioxidant enzyme activity, and maintenance of tissue homeostasis (Lebeer et al., 2008). Recent evidence suggests that certain probiotic strains can activate the NRF2/ARE signaling pathway, thereby enhancing cellular antioxidant defenses and providing protection against oxidative damage (Jones et al., 2015).

Despite growing concerns about NPs’ exposure and their health implications, limited research has investigated the effects of these particles on salivary gland function or explored potential protective interventions. Furthermore, to our knowledge, no studies have examined the protective potential of Pro against NPs-induced toxicity in oral tissues. Given the critical role of salivary glands in oral health maintenance and their vulnerability to environmental contaminants, understanding the mechanisms of nanoplastic toxicity and identifying effective protective strategies is of paramount importance.

Probiotics have been reported to exert biological effects beyond the gastrointestinal tract by modulating systemic immune and redox signaling pathways. Strains belonging to the *Lactobacillus* and Bifidobacterium genera possess antioxidant and anti-inflammatory properties and can regulate key pathways involved in oxidative stress. In particular, probiotic supplementation has been shown to activate the NRF2 signaling pathway and increase the expression of downstream antioxidant enzymes such as HO-1, thereby enhancing cellular antioxidant defenses (Aboulgheit et al., 2021). Moreover, probiotics may attenuate inflammatory responses by inhibiting the NF-κB signaling pathway (Niu et al., 2024). These systemic effects are thought to occur through the gut–organ axis, where microbial metabolites and immune mediators influence distant tissues beyond the intestine (Plaza-Diaz et al., 2019). Accordingly, probiotic supplementation has been shown to reduce oxidative stress and inflammation in various experimental models (Liu et al., 2018).

Therefore, the present study aimed to investigate the effects of polystyrene nanoplastics on rat parotid glands and evaluate the potential protective role of multi-strain probiotic supplementation. We hypothesized that NPs exposure would induce oxidative stress, inflammatory responses, and tissue damage in parotid glands, while probiotic treatment would mitigate these adverse effects through activation of antioxidant defense pathways and suppression of inflammatory cascades. The findings of this research may provide valuable insights into the mechanisms of NPs toxicity in oral tissues and establish a foundation for developing probiotic-based therapeutic strategies to protect against environmental pollutant-induced health risks.

## Materials and methods

### Ethics statement

This experimental study was conducted in accordance with the Erzincan Binali Yıldırım University Animal Experiments Local Ethics Committee guidelines and was approved by the committee (Decision No. 32, Doc. No. E-85748827-804.99-468589). All procedures adhered to the principles of the Declaration of Helsinki for animal research and to institutional guidelines for the care and use of laboratory animals.

### Experimental animals

Forty adult male Sprague-Dawley rats weighing 250–300 g were obtained from the Erzincan Binali Yıldırım University Animal Research and Application Center (EBYÜ-DEHAM). Animals were housed under standard laboratory conditions with controlled temperature (20 °C–26 °C), humidity (40%–60%), and 12:12-h light-dark cycles. Rats received standard laboratory chow and water *ad libitum*. All animals underwent a 7-day acclimatization period prior to experimental procedures.

### Experimental design and groups

Rats were randomly allocated into five experimental groups (n = 8 per group) as follows:

Control group (n = 8): Oral administration of normal saline via gavage for 35 days.

Pro (10^10^) group (n = 8): Probiotic mixture (10^10^ CFU/day) administered orally for 35 days (Liong, 2008).

NPs group (n = 8): Polystyrene nanoplastics (10 mg/kg body weight) administered orally via gavage for 35 days (Ma et al., 2023).

Pro (10^9^) + NPs group (n = 8): NPs (10 mg/kg) + probiotic mixture (10^9^ CFU/day) administered orally for 35 days.

Pro (10^10^) + NPs (n = 8): NPs (10 mg/kg) + probiotic mixture (10^10^ CFU/day) administered orally for 35 days.

All treatments were administered once daily at approximately the same time (09:00-10:00 AM) for 35 consecutive days.

### Polystyrene nanoplastics (NPs) materials and preparation

Commercially available polystyrene nanoparticles (NPs; 100 nm diameter; Sigma-Aldrich Co., Cat. no: 43302) were used in this study. According to the manufacturer’s specifications, the particles are supplied as a 10% (w/v) solids aqueous suspension with a density of approximately 1.05 g/cm^3^. Detailed physicochemical characteristics, including particle size distribution, surface properties, and morphology, are provided by the manufacturer in the official product documentation and Certificates of Analysis available online.

Stock solutions were prepared by dispersing NPs in sterile distilled water and then ultrasonicated for 15 min to prevent aggregation. Working solutions were freshly prepared daily and administered at a dose of 10 mg/kg body weight in 0.3 mL. This dose was selected based on established experimental protocols in the NPs toxicology literature; Yasin et al. (2022) employed the same dose (10 mg/kg) and duration (35 days) to evaluate NPs-induced toxicity in a rat model, and similar doses have been widely used across multiple organ-system studies to produce detectable biological effects within feasible study durations while remaining within a range that allows extrapolation to chronic human exposure scenarios.

### Multi-strain probiotic preparation

A commercially available multi-strain probiotic formulation (Enzibody®, Kenz BioTech, United States) was used in this study. To ensure experimental precision and batch consistency, each of the 12 bacterial strains was obtained from the manufacturer as separate, individual isolates rather than as a pre-mixed compound. These strains included: *Bifidobacterium infantis, Lactobacillus acidophilus, Bifidobacterium thermophilum, Lactobacillus casei, Bifidobacterium longum, Lactobacillus helveticus, Lactobacillus plantarum, Lactococcus lactis, Leuconostoc mesenteroides, Lactobacillus paracasei, Lactobacillus bifidus,* and *Lactobacillus brevis*.

Each strain was cultured independently in Man, Rogosa, and Sharpe (MRS) broth and incubated overnight at 37 °C under standardized anaerobic conditions. After incubation, the concentration of each separate culture was determined using optical density (OD) measurements, which were pre-calibrated against colony-forming unit (CFU) standards for each specific strain. Based on these measurements, each bacterial suspension was adjusted to an equivalent concentration of approximately 8.33 x times 10^10 CFU/mL.

Following concentration adjustment, bacterial cells were harvested by centrifugation at 3000 *g* for 15 min, washed three times with ice-cold phosphate-buffered saline (PBS, pH 7.2), and resuspended. Equal volumes of these standardized suspensions were then combined to obtain a final multi-strain probiotic mixture with a total bacterial concentration of 1 × 10^12 CFU/mL. For experimental administration, working suspensions (1 x times 10^10 and 1 x times 10^9 CFU/day) were prepared daily by diluting the stock in PBS. To ensure maximum viability, all doses were stored at 4 °C and administered within 2 h of preparation. This rigorous protocol was employed to guarantee the exact contribution of each strain to the final formulation and to ensure experimental reproducibility.

### Sample collection and processing

After 35 days of treatment, all animals were fasted for 12 h before sample collection. Rats were anesthetized with an intraperitoneal injection of xylazine (4 mg/kg; Rompun, Bayer) and ketamine (40 mg/kg; Ketalar, Pfizer). Blood samples (5–7 mL) were collected via cardiac puncture and centrifuged at 4000 rpm for 10 min at 4 °C. Serum was separated and stored at −20 °C for biochemical analyses. During the experimental period, no mortality was recorded in any of the groups. At the end of the study, a systematic necropsy was performed on all animals to assess general health and to exclude any gross pathological findings beyond the parotid gland.

Following blood collection, animals were euthanized by exsanguination under deep anesthesia. Parotid glands were carefully dissected and divided into two portions: one portion was immediately fixed in 10% neutral-buffered formalin for histopathological examination, while the remaining tissue was snap-frozen in liquid nitrogen and stored at −80 °C for biochemical and Western blot analyses.

### Biochemical analyses

#### Measurement of lipid peroxidation and antioxidant enzyme activities and levels

Parotid salivary gland tissues were mechanically homogenized in 1 mL phosphate-buffered saline (PBS, pH 7.4) on ice, without the use of a chemical lysis buffer. The homogenates were centrifuged at 10,000 × g for 15 min at 4 °C, and the supernatants were collected for biochemical analyses. Protein concentration was determined using the Bradford method based on Coomassie Brilliant Blue G-250 dye binding (Bradford, 1976). Bovine serum albumin (BSA; MedChemExpress, United States) was used as the standard protein for calibration. A standard calibration curve was generated using serial dilutions of BSA in the range of 0–1000 μg/mL, and absorbance was measured at 595 nm using a microplate reader. Malondialdehyde (MDA) levels in salivary gland supernatants (Cat. no. E0156Ra, BT-LAB) were measured using commercial ELISA kits according to the manufacturer’s instructions. Similarly, superoxide dismutase (SOD) activity (Cat. no. E0168Ra, BT-LAB) and glutathione (GSH) levels (Cat. no. E1101Ra, BT-LAB) were analyzed using commercial ELISA kits following the manufacturer’s protocols. All biochemical parameters were normalized to total protein content and expressed per mg protein.

### Histopathological analysis

Parotid gland tissues from rats in the experimental groups were collected during systemic necropsy and immediately fixed in 10% neutral-buffered formalin for 72 h. After fixation, tissues were dehydrated through a graded alcohol series (50%, 70%, 80%, 96%, and 100%), cleared in xylene, and embedded in paraffin. Sections of 5 µm thickness were obtained from paraffin blocks using a microtome (RM2125 RTS) and mounted on glass slides. The sections were stained with Mallory’s trichrome staining method modified by Crossman and examined under a light microscope (Nikon Eclipse E600). Representative images were captured at 200× magnification.

Histopathological evaluation was performed using a semi-quantitative scoring system. For each section, five randomly selected microscopic fields were examined under a 20× objective lens. The evaluated parameters included degenerated acini, degenerative changes in acinar cells, and the degree of fibrosis within the parenchyma–stroma. The severity of the histopathological findings was graded as follows: absent (−), mild (+), moderate (++), and severe (+++). The arithmetic means of the scores obtained from the examined fields was calculated to determine the final histopathological score for each specimen.

All histopathological assessments were performed in a blinded manner by a trained observer using standardized and previously described histopathological evaluation criteria (Bancroft and Gamble, 2008; Suvarna et al., 2018). To minimize potential observational bias, all samples were analyzed under identical experimental conditions, including the same microscope, magnification, and evaluation protocol, ensuring methodological consistency and reproducibility across the experimental groups.

### Western blot analysis

Western blot analysis was used to examine changes in protein expression in rat parotid gland tissue samples. Antibodies specific to rats were used as listed in Table 1. Serum proteins were separated by SDS–PAGE and transferred onto PVDF membranes (Ecotech Biotech., Türkiye) using a semi-dry ‘Turbo blotter’ system with an 8-layer filter paper stack. Due to the number of samples, multiple gels and membranes were used, and representative lanes were cropped for figure presentation. All gels and membranes were processed under identical experimental conditions, and β-actin was used as a common internal loading control to normalize target protein expression levels.

**TABLE 1:** Antibodies used in Western blot analysis and their manufacturers.

Protein	Manufacturers
Caspase-3	Santa Cruz, sc- 56,053
Bcl-2	Affinity Biotech, AF4650
HO-1	Santa Cruz Sc-10789
NRF2	Santa Cruz Sc-365949
NF-κB p65	Santa Cruz, Sc-8008
IL-1β	Santa Cruz, sc-12742
TNF-α	Santa Cruz sc-52746
AQP5	Santa Cruz, sc-514022
Beta-actin	Santa Cruz, sc-47778
Secondary antibody (Goat anti-rabbit IgG-HRP)	Santa Cruz, sc-2004
Secondary antibody (Mouse anti-rabbit IgG-HRP)	Santa Cruz, sc-2357

Following transfer, PVDF membranes were incubated in blocking buffer for 1 h, and primary antibodies (5 μg/mL) were added and incubated overnight at 4 °C on an orbital shaker. After five washes in PBS-T (5 min each), secondary antibodies (1 μg/mL) were added for 1 h at room temperature, followed by another five washes. Chemiluminescent HRP substrate (Ecotech Biotech., Türkiye) was applied, and the blots were imaged using a gel imaging system (Bio-Rad Imaging System). Band intensities were quantified using Image Lab software (Bio-Rad, United States). Total protein normalization was used for protein expression analysis. This approach ensured consistency and reliability across multiple gels and membranes by correcting for loading variability and technical differences while maintaining experimental reproducibility.

### Statistical analysis

All data are expressed as mean ± standard deviation (SD). Statistical analyses were performed using SPSS software (version 25.0; IBM Corp., Armonk, NY, United States). Prior to statistical comparisons, the normality of data distribution was assessed using the Shapiro–Wilk test. For data showing normal distribution, comparisons among multiple groups were performed using one-way analysis of variance (ANOVA) followed by Tukey’s honestly significant difference (HSD) post-hoc test for pairwise comparisons. For variables that did not meet the assumption of normality, the non-parametric Kruskal–Wallis test was applied. A p-value <0.05 was considered statistically significant. Each experimental group consisted of eight animals (n = 8), which was considered sufficient to provide adequate statistical power for detecting biologically meaningful differences between groups.

## Results

### Oxidative stress biomarkers

Significant differences were observed in oxidative stress parameters among the experimental groups in parotid gland tissues (p < 0.05).

**FIGURE 1:**
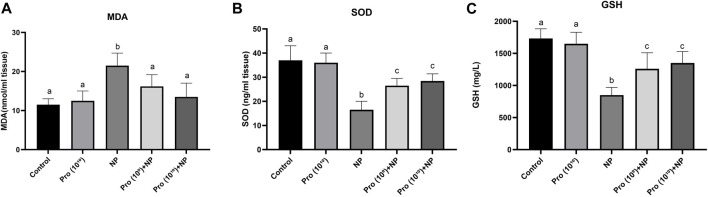
Levels of oxidative stress markers in parotid salivary gland tissues across experimental groups. **(A)** Malondialdehyde (MDA), **(B)** superoxide dismutase (SOD), and **(C)** glutathione (GSH). Exposure to polystyrene nanoplastics (NPs) increased MDA levels, indicating enhanced lipid peroxidation, while significantly decreasing the antioxidant markers SOD and GSH compared with the control group. Data are presented as mean ± standard deviation (SD) for each group (n = 8). Different letters above the bars (a, b, c, ab) indicate statistically significant differences between groups (p < 0.05). Statistical comparisons were performed using one-way analysis of variance (ANOVA) followed by Tukey’s post hoc test.

NPs administration significantly increased MDA levels (p < 0.05), indicating enhanced lipid peroxidation, as MDA is a widely recognized marker of oxidative damage formed via peroxidation of polyunsaturated fatty acids. As shown in Figure 1A, the NPs group exhibited markedly elevated MDA concentrations compared with controls. Both NPs + Probiotics 10^9^ CFU and NPs + Probiotics 10^10^ CFU groups showed a trend toward reduced MDA levels compared to NPs alone (p < 0.05).

SOD activity was significantly lower in the NPs group than in controls, indicating compromised antioxidant enzyme capacity. As demonstrated in Figure 1B, NPs-treated animals exhibited notably reduced SOD levels compared to the control group. Notably, both NPs + Probiotics 10^9^ CFU and NPs + Probiotics 10^10^ CFU groups demonstrated significant recovery of SOD activity (p < 0.05), with levels approaching those observed in control animals.

GSH, considered the most abundant endogenous antioxidant molecule, showed significant depletion in NPs-exposed animals. As illustrated in Figure 1C, GSH levels were markedly reduced in the NPs group compared with controls. Probiotic supplementation significantly ameliorated this reduction, with both treatment groups showing restored GSH levels comparable to controls (p < 0.05).

### Histopathological findings

Microscopic examination revealed distinct histopathological changes in parotid gland architecture following NPs exposure.

**FIGURE 2:**
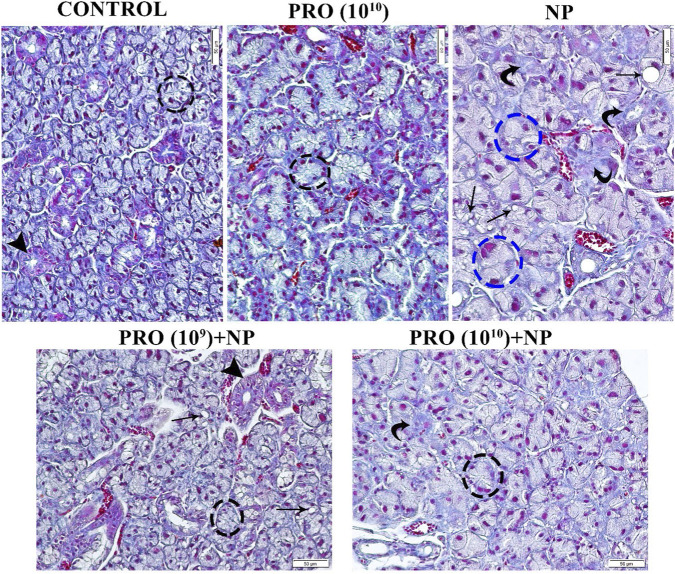
Histopathology of rat parotid gland tissue, Crossman’s Modified Mallory’s Triple Stain, ×200 magnification, scale bar = 50 µm. Arrowhead: Secretory lobe; Black circle: Serous acinus; Blue circle: Degenerative acinus; Curved arrow: Fibrosis; Arrow: Vacuolization in acinar cells. Each group included n = 8 rats, and all animals were analyzed. No samples were excluded during the study.

Both the control and probiotics-alone (10^10^ CFU) groups exhibited normal histological appearance with well-preserved acinar architecture. Serous acini showed typical eosinophilic cytoplasm with basally located nuclei, and no degenerative changes were observed, as shown in Figure 2.

Substantial histopathological alterations were evident in the NPs group, as demonstrated in Figure 2. The parotid gland tissue showed moderate-to-severe acinar degeneration (++) and prominent cellular vacuolization (++). Additionally, there was increased connective tissue deposition, indicating fibrosis (++), accompanied by loss of normal acinar architecture.

Both treatment groups (10^9^ and 10^10^ CFU) demonstrated marked improvement compared to NPs alone, as illustrated in Figure 2. The NPs + Probiotics 10^9^ CFU group showed mild acinar degeneration (+) and mild vacuolization (+) with minimal fibrosis (−). Similarly, the NPs + Pro 10^10^ CFU group exhibited mild acinar degeneration (+), no vacuolization (−), and mild fibrosis (+). These findings suggest that probiotic supplementation provides significant protection against NPs-induced structural damage.

Semi-quantitative analysis confirmed significant protective effects of probiotic supplementation against NPs-induced structural damage (Table 2).

**TABLE 2:** Semi-quantitative analysis of parotid gland histopathology.

Group	Degenerative acini	Cellular vacuolization	Acidophilic cytoplasm	Fibrosis
Control	-	-	-	-
Pro (10^10^)	-	-	-	-
NPs	++	++	-	++
NPs-pro (10^9^)	+	+	-	-
NPs-pro (10^10^)	+	-	-	+

Scoring: (-) absent, (+) mild, (++) moderate, (+++) severe.

### Western blot analysis

Protein expression analysis revealed significant alterations in key cellular pathways following NPs exposure, including apoptosis, oxidative stress response, and inflammation, consistent with mechanisms of nanoplastic-induced cellular damage.

### Apoptosis-related proteins

**FIGURE 3:**
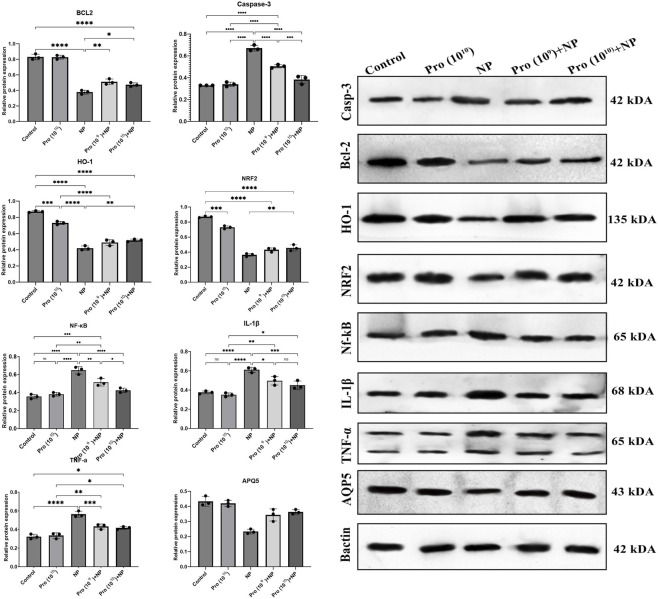
Effect of Pro (10^9^ and 10^10^ CFU/mL) and nanoplastics (NP) applications on apoptosis (Bcl-2, Casp-3), oxidative stress (HO-1, NRF2), inflammation (NF-κB, IL-1β, TNF-α), and aquaporin 5 (AQP5) protein levels in rat tissues. β-actin was used as an internal control. Data are expressed as mean ± SD (n = 3 per group). Statistical analysis was performed using ANOVA (*p < 0.05, **p < 0.01, ***p < 0.001, ****p < 0.0001). No animals were excluded; attrition rate was zero.

Analysis of apoptotic markers revealed significant dysregulation following NPs exposure. Anti-apoptotic Bcl-2 protein showed significant downregulation in NPs-treated animals compared to controls (p < 0.01), indicating activation of apoptotic pathways, as demonstrated in Figure 3. Probiotics (10^9^ CFU) alone significantly increased Bcl-2 expression above control levels (p < 0.0001), while both NPs + Probiotics groups demonstrated significant recovery of Bcl-2 levels compared to NPs alone (10^9^: p < 0.05; 10^10^: p < 0.01). Conversely, pro-apoptotic caspase-3 was markedly upregulated in the NPs group compared with controls (p < 0.0001), confirming activation of apoptotic cell death pathways, as shown in Figure 3. Both probiotic groups-maintained caspase-3 levels similar to controls and significantly lower than those in NPs alone (p < 0.01), and the NPs + Probiotics group also showed a significant reduction compared with NPs (p < 0.01).

### Oxidative stress response proteins

The antioxidant defense system was severely compromised following NPs exposure. Heme oxygenase-1, a key cytoprotective enzyme, was severely depleted in the NPs group compared to controls (p < 0.0001), as illustrated in Figure 3. Both probiotic treatments significantly increased HO-1 levels above controls (p < 0.001), and the NPs + Probiotics groups showed significant recovery compared with NPs alone (p < 0.01). Similarly, nuclear factor erythroid 2-related factor 2, a master regulator of antioxidant response, was significantly decreased in NPs group (p < 0.01), as demonstrated in Figure 3. Probiotics (10^9^) maintained levels near controls, while probiotics (10^10^) exceeded control levels (p < 0.01). Both NPs + Probiotics groups showed significant NRF2 recovery (p < 0.01), indicating restoration of cellular antioxidant capacity.

### Inflammatory markers

NPs exposure triggered robust inflammatory responses through multiple pathways. Nuclear factor-κB, a key inflammatory transcription factor, showed significant upregulation in NPs group (p < 0.001), indicating activation of inflammatory pathways, as shown in Figure 3. Probiotic treatments effectively suppressed this elevation, and the NPs + Probiotics groups demonstrated a significant reduction compared with NPs (p < 0.01), approaching control levels. Pro-inflammatory cytokines were similarly affected, with interleukin-1β showing a marked elevation in the NPs group compared with controls (p < 0.001), as illustrated in Figure 3. Both probiotics groups-maintained IL-1β levels similar to controls, while NPs + Probiotics groups showed a significant reduction compared to NPs alone (p < 0.01). Tumor necrosis factor-α demonstrated a similar pattern with a significant increase in the NPs group (p < 0.001), as demonstrated in Figure 3. Probiotics (10^10^) maintained levels similar to controls and showed a significant reduction versus NPs (p < 0.05), with the NPs + Probiotics (10^10^) group also showing a significant decrease compared to NPs (p < 0.001).

### Functional protein expression

The functional integrity of parotid glands was assessed through aquaporin-5 expression, which is essential for salivary gland function. AQP5 was severely reduced in the NPs group compared to controls (p < 0.0001), as shown in Figure 3, indicating compromised glandular function. Both probiotic groups showed AQP5 levels above those of controls, suggesting enhanced functional capacity. NPs + Probiotics groups demonstrated significantly greater recovery than NPs (p < 0.001), indicating restoration of salivary gland function. All protein expression data and corresponding band densities are presented in Figure 3.

## Discussion

Polystyrene nanoplastics represent emerging environmental contaminants that pose significant threats to human health through their ability to penetrate biological barriers and accumulate in various tissues (Lehner et al., 2019). Parotid glands, as the largest salivary glands, play crucial roles in maintaining oral homeostasis through saliva production and are particularly vulnerable to oxidative damage due to their high metabolic activity and exposure to environmental toxins (Proctor, 2000). The present study, to our knowledge the first to demonstrate this, showed that probiotic supplementation provides significant protection against polystyrene nanoplastic-induced oxidative stress and tissue damage in rat parotid glands. Our findings reveal that PS-NP exposure elicits comprehensive pathological changes, including enhanced lipid peroxidation, depleted antioxidant defenses, inflammatory activation, and structural alterations, whereas multi-strain probiotic intervention effectively mitigates these deleterious effects by modulating key cellular protective pathways.

In salivary glands, oxidative stress disrupts normal secretory function, impairs cellular metabolism, and triggers inflammatory cascades that compromise tissue integrity (Tóthová et al., 2015). Our results align with emerging evidence demonstrating that nanoplastic exposure induces systemic oxidative stress across various organ systems. Milillo et al. (2024) reported that NPs with a diameter of 800 nm significantly affected cell viability, inducing oxidative stress, cellular senescence, and apoptosis in human alveolar epithelial cells. Similarly, Ali et al. (2024) found that exposure to micro- and nanoplastics (MNPs) can lead to health effects through oxidative stress, inflammation, immune dysfunction, altered biochemical and energy metabolism, impaired cell proliferation, disrupted microbial metabolic pathways, abnormal organ development, and carcinogenicity. The significant elevation in MDA levels and depletion of SOD and GSH observed in our NPs-exposed parotid glands corroborate these findings.

Nanoplastic-induced mitochondrial dysfunction represents a critical mechanism underlying cellular toxicity, characterized by compromised energy production, altered membrane potential, and increased reactive oxygen species generation (Brand, 2016). Recent studies have shown that polystyrene nanoparticles also induced mitochondrial dysfunction, as evidenced by membrane changes and reduced cellular energy metabolism, and activated endoplasmic reticulum stress, as demonstrated by increased endoplasmic stress markers (Osman et al., 2023). The mechanism of NPs-induced oxidative stress appears to involve disruption of cellular redox homeostasis through multiple pathways. Liang et al. (2021) demonstrated that exposure to PS micro- and nanoplastics was correlated with ROS generation, epithelial cell apoptosis, and increased intestinal permeability, suggesting that nanoplastics may exert systemic effects by modulating cellular communication networks.

In this context, the histopathological alterations and biochemical changes observed in the parotid gland in the present study may be partly attributed to oxidative stress-mediated mitochondrial dysfunction and inflammation triggered by PS-NP exposure. Multi-strain probiotics may counteract these adverse effects through several mechanisms, including modulation of oxidative stress responses, enhancement of antioxidant defense systems, and regulation of inflammatory signaling pathways. In addition, probiotics may help maintain cellular homeostasis by improving epithelial barrier integrity and modulating microbiota-host interactions, thereby reducing systemic toxicity induced by nanoplastic exposure.

Nuclear factor erythroid 2-related factor 2 (NRF2) functions as the master regulator of cellular antioxidant defense, controlling the transcription of numerous cytoprotective genes including antioxidant enzymes, detoxification proteins, and stress response factors (Cuadrado et al., 2019). The observed reduction in NRF2 expression in our study is particularly significant, as NRF2 is a transcription factor that robustly transduces chemical signals to regulate a battery of cytoprotective genes and represents the master regulator of cellular antioxidant defense (Yamamoto et al., 2018; Mutluay et al., 2025). This finding is consistent with Wang et al. (2024), who reported that NPs modified by different functional groups caused a notable rise in fasting blood glucose levels, glucose intolerance, and insulin resistance, along with glycogen accumulation and hepatocellular edema, indicating widespread metabolic disruption.

Chronic inflammation in glandular tissues involves complex molecular cascades including nuclear factor-κB activation, pro-inflammatory cytokine release, and immune cell recruitment, ultimately leading to tissue damage, fibrosis, and functional impairment (Medzhitov, 2008). In the parotid glands, inflammatory processes disrupt normal acinar architecture, compromise secretory capacity, and trigger apoptotic pathways, resulting in progressive tissue degeneration (Buyuk et al., 2015). The marked upregulation of NF-κB, IL-1β, and TNF-α in NPs-exposed animals reflects activation of pro-inflammatory cascades. A recent systematic review has demonstrated that nanoplastic exposure induces oxidative stress, cytokine imbalance, and activation of pro-inflammatory pathways, resulting in tissue-specific cellular damage across multiple organs (Skaba et al., 2025). This inflammatory response contributes to the histopathological changes observed, including acinar cell degeneration, vacuolization, and fibrosis development.

Apoptotic cell death is a programmed cellular response to severe stress, characterized by activation of caspase cascades, mitochondrial dysfunction, and, ultimately, cell elimination to prevent tissue damage (Green and Llambi, 2015; Gür et al., 2022). The concurrent elevation of caspase-3 and reduction of Bcl-2 expression indicate activation of apoptotic pathways, supporting the concept that 100 nm MP are internalized by intestinal epithelial cells via endocytosis, triggering oxidative stress and ferroptosis (Cheng et al., 2025). Studies in aquatic animals have shown that 14-day exposure to 100 μg/L of polystyrene nanoplastics resulted in significant alterations in lipid metabolism and antioxidant systems (Okon et al., 2025), suggesting size-dependent toxicity mechanisms in which nanoscale particles may be particularly harmful due to their enhanced cellular uptake.

Probiotics are live microorganisms that confer health benefits through multiple mechanisms, including immune modulation, activation of antioxidant enzymes, and maintenance of tissue homeostasis when administered in adequate amounts (Hill et al., 2014). In the context of environmental toxin exposure, probiotics can enhance cellular defense mechanisms, reduce inflammatory responses, and promote tissue repair processes (Kechagia et al., 2013). To our knowledge, this is the first study to demonstrate the protective effects of multi-strain probiotic supplementation against NPs toxicity, representing a novel therapeutic approach for nanoplastic-induced tissue damage. Recent reviews have highlighted that probiotic bacteria could be considered for both prevention and treatment of microplastic/nanoplastic-induced disorders, as they can modulate various protective pathways simultaneously (Bazeli et al., 2023). Recent studies using engineered probiotics have shown that they can mitigate gut barrier dysfunction induced by nanoplastics (Chen et al., 2025), supporting our findings in salivary gland tissue.

The NRF2/HO-1 pathway is the primary cellular defense mechanism against oxidative stress, involving the nuclear translocation of the NRF2 transcription factor and the subsequent upregulation of antioxidant enzymes, including heme oxygenase-1, superoxide dismutase, and glutathione synthesis enzymes (O’Rourke et al., 2024). Probiotics significantly enhanced NRF2 and HO-1 expression, key components of the cellular antioxidant response system. Wati et al. (2020) (12) demonstrated that NRF2 activation by KEAP1 inhibition attenuated the manifestation of aging phenotypes in salivary glands by reducing oxidative stress markers, consistent with our observed probiotic-mediated protection. Lactobacilli cell-free supernatants have been shown to modulate inflammation and oxidative stress in human microglia via NRF2-SOD1 signaling (Di Chiano et al., 2024), demonstrating direct activation of antioxidant pathways by bacterial metabolites.

Glutathione and superoxide dismutase systems constitute essential components of cellular antioxidant defense, with glutathione serving as the primary intracellular reducing agent and SOD catalyzing the dismutation of superoxide radicals to hydrogen peroxide and oxygen (Forman et al., 2009). In our study, the restoration of SOD and GSH levels in the PS-NP + Probiotic groups suggests an activation of the NRF2-mediated antioxidant response. The KEAP1/NRF2/ARE signaling pathway primarily regulates the expression of antioxidant and anti-inflammatory genes, including HO-1, SOD, and GSH (Ahmed et al., 2017; Loboda et al., 2016). Jones et al. (2015) previously demonstrated that *Lactobacillus* GG modulates epithelial cytoprotection through the Nrf2 pathway in a species- and strain-specific manner.

Interestingly, we observed that SOD and GSH levels in the probiotic-only groups were slightly lower than those in the control group. This can be attributed to a decreased requirement for endogenous antioxidant mobilization. Since probiotics can directly neutralize reactive oxygen species (ROS) through their own metabolites and maintain systemic redox stability, they may reduce the immediate physiological demand on the host’s enzymatic defense systems in a non-stressed (control) state. This suggests that probiotics optimize the redox environment rather than overstimulating it.

Nuclear factor-κB represents a central transcriptional regulator of inflammatory responses, controlling the expression of pro-inflammatory cytokines, chemokines, and adhesion molecules in response to cellular stress and pathogen-associated molecular patterns (Liu et al., 2017). The suppression of NF-κB, IL-1β, and TNF-α expression by probiotic treatment reflects potent anti-inflammatory activity. Several studies have demonstrated that Nrf2 contributes to the anti-inflammatory process by orchestrating the recruitment of inflammatory cells and regulating gene expression through the ARE (Ma, 2013). Recent studies have shown that probiotics activate antioxidant mechanisms and can suppress extensive oxidative stress via their ability to activate Nrf2 (Karaca et al., 2022).

Crosstalk between antioxidant and inflammatory pathways involves complex molecular interactions where NRF2 activation can suppress NF-κB-mediated inflammatory responses while NF-κB signaling can influence antioxidant gene expression (Bellezza et al., 2018). This anti-inflammatory mechanism appears to involve complex crosstalk between antioxidant and inflammatory pathways. The NRF2 transcription factor modulates the expression of defense genes encoding detoxifying enzymes and antioxidant proteins, while also influencing NF-κB-mediated inflammatory responses (Kensler et al., 2007). Our findings demonstrate that probiotics can effectively modulate this crosstalk, thereby enhancing antioxidant defenses and suppressing inflammatory responses.

Aquaporin-5 serves as the primary water channel protein in salivary gland acinar cells, facilitating rapid water transport across cellular membranes and playing essential roles in saliva production, composition, and secretory function (Hosoi, 2016). The recovery of AQP5 expression in probiotic-treated animals suggests restoration of salivary gland functionality. AQP5 is essential for water transport and saliva production, and its downregulation following NPs exposure indicates compromised glandular function. The ability of probiotics to restore AQP5 levels suggests that these beneficial microorganisms can preserve not only cellular integrity but also organ-specific functional capacity.

## Conclusion

Multi-strain probiotic supplementation represents a novel therapeutic approach to protect against environmental nanoplastic toxicity through multiple mechanisms, including activation of antioxidant pathways, anti-inflammatory effects, and preservation of tissue function. This study, to our knowledge, provides the first evidence that multi-strain probiotic supplementation offers significant protection against NPs-induced toxicity in salivary glands. The protective mechanisms involve restoration of NRF2/HO-1-mediated antioxidant defenses, suppression of NF-κB-driven inflammatory responses, inhibition of apoptotic pathways, and preservation of glandular function. However, certain limitations should be acknowledged: direct assessment of probiotic colonization efficiency (fecal bacterial enumeration, 16S rRNA sequencing) and independent physicochemical characterization of NPs were not performed in the present study and should be addressed in future investigations. These findings suggest that probiotics represent a promising therapeutic strategy for mitigating the adverse health effects of environmental nanoplastic exposure, with potential applications extending beyond oral health to systemic protection against plastic pollution-induced toxicity.

## Data Availability

The datasets used and/or analyzed during the current study are available from the corresponding authors upon reasonable request.
